# DynaMed

**DOI:** 10.29173/jchla29848

**Published:** 2025-12-01

**Authors:** Mackenzie Hilton

**Affiliations:** Librarian Centre for Addiction and Mental Health (CAMH) Toronto, ON, Canada

**Product:** DynaMed

**URL:** https://www.dynamed.com/

## Purpose

The purpose of this product review is to elucidate the strengths and weaknesses of DynaMed. A comparative analysis will be made between DynaMed and BMJ Best Practice to serve as a guide to help health sciences libraries decide which point-of-care tool best suits their users’ needs.

## Product description

“DynaMed is a clinical decision support solution with a mission to provide the most useful evidence-based information to healthcare professionals at the point-of-care” [1]. Content in DynaMed is based on a “rigorous, seven-step evidence-based methodology, robust systematic literature surveillance with daily updates, and clinical expertise that both complements and clarifies the evidence” [2].

## Intended users

DynaMed is a clinical reference tool intended for physicians, nurses, allied health, and other health care professionals for use primarily at the point-of-care. However, DynaMed is useful for anyone looking for background information or additional resources on clinical topics.

## Special features

### Condition overviews

**Fig. 1:**
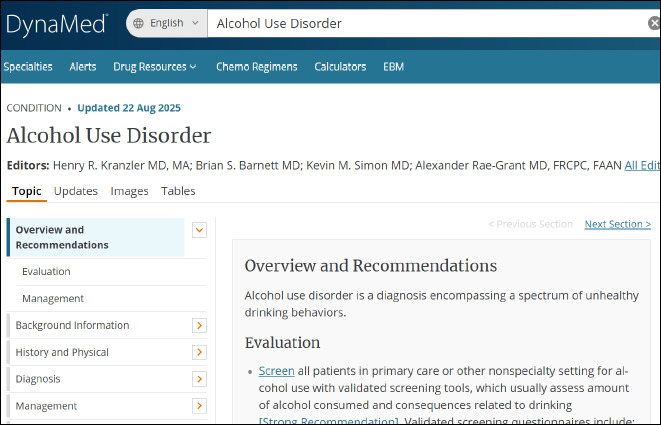
Overview and recommendations for alcohol use disorder

Users can read topic overviews and recommendations, including recommendations for the evaluation and management of specific conditions (Figure 1).

Users can navigate between tabs to view information regarding page updates, images, tables, and editors and reviewers.

### Specialties

**Fig. 2:**
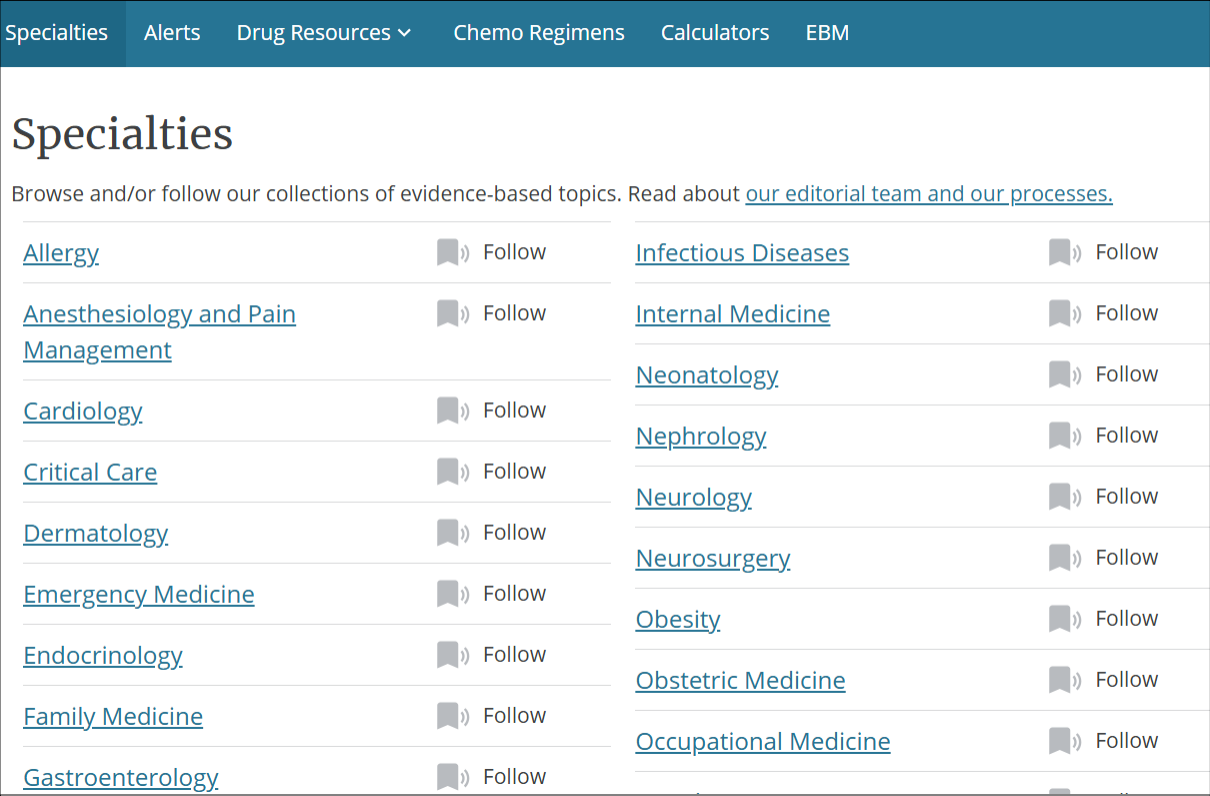
Speciality topics in DynaMed

Navigating to the “Specialties” page (Figure 2) allows users to select from 45 different specialties. Specialties in DynaMed serve as collections of evidence-based topics. For example, users can select the specialty “Substance Use and Addiction Medicine” and view several narrower topics, including “Conditions and Behaviors,” “Substance Use in Specific Populations,” and “Complications.”

### Recent alerts

Alerts inform users of recent content updates made in DynaMed. Recent alerts can be viewed directly on the home screen and can be personalized after creating an account. Alerts also include links to the topics where the updates are made. Additional features, such as the ability to filter alerts by categories, are accessed by navigating to the “Alerts” page. Filtering by categories allows users to select from 42 “Topic” categories. Users can select as many categories to filter as they wish by checking the relevant boxes.

### Drug monographs from Micromedex

Users can view information about drugs and medications. Drug monographs are organized alphabetically. Users can search for specific drugs using the search bar. Using the drug Naltrexone as an example, information is organized into the following categories:

- Dosing/Administration
- Medication Safety
- Class
- Mechanism of Action
- Pharmacokinetics
- Patient Education
- Toxicology
- About
- Brands

### Drug interactions

**Fig. 3:**
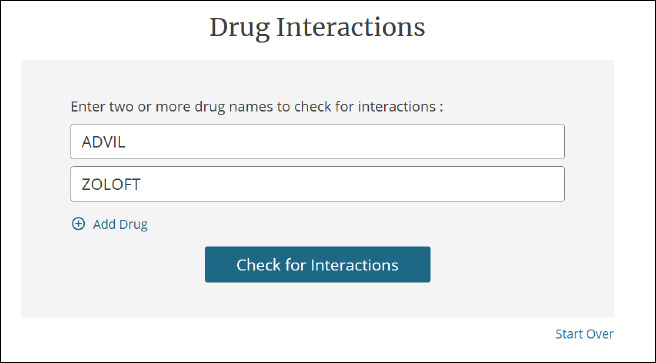
Checking for drug interactions between Advil and Zoloft

Users can compare two or more drugs to check for interactions (Figure 3). Users can search for the trade name (e.g. Tylenol) or generic name of the drug (e.g. acetaminophen). Results can be filtered by:

- Drug/ethanol interactions
- Drug/food interactions
- Drug/ingredient duplication interactions
- Drug/lab interactions
- Drug/lactation interactions
- Drug/pregnancy interactions
- Drug/tobacco interactions

### Calculators

**Fig. 4:**
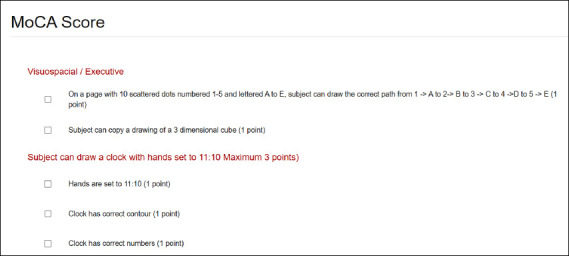
A MoCA score calculator

DynaMed also contains a variety of calculators (Figure 4), questionnaires, screening tools, decision trees, and scales. These tools can be used for a variety of purposes, including converting dosages, calculating statistics, and calculating nutritional values.

### Chemotherapy regimens

**Fig. 5:**
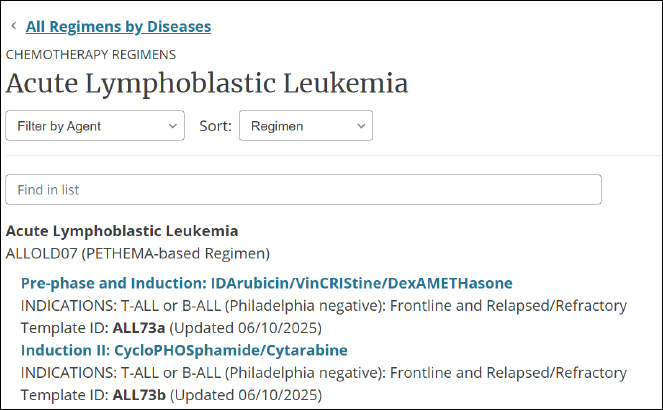
Chemotherapy regimens for acute lymphoblastic leukemia

“[DynaMed] contains 2500+ peer-reviewed chemotherapy regimens from the National Comprehensive Cancer Network (NCCN) describing currently accepted treatments that help ensure the safe use of drugs and biologics in cancer care” [3]. Users can sort information by disease (e.g. breast cancer; Kaposi sarcoma), and by agent (e.g. Alectinib; eriBULin) (Figure 5).

## Compatibility

DynaMed can be accessed via a web browser or mobile application. Compatible web browsers include Google Chrome, Firefox, and Microsoft Edge. On June 15, 2022, DynaMed stopped supporting Microsoft’s Internet Explorer [4]. The DynaMed app is available on iOS and Android devices.

## Usability

DynaMed is easy to search and use. DynaMed includes a basic search, which simplifies the searching process for users. The search functionality in DynaMed does not have the option for advanced search functions, such as Boolean operators, truncation, and adjacency modifiers. EBSCO provides “How-To Information” and tutorial videos that teach users how to use and search DynaMed.

## Costs

DynaMed requires a subscription to access. The price of a 1-year individual subscription ranges from USD $136-546, depending on the user’s profession (e.g. students, residents, licensed practitioners, and physicians) [5]. Institutional subscriptions are also available. The cost of an institutional subscription can vary widely between institutions. Prospective users can ask a DynaMed representative to learn how much an institutional subscription will cost for them.

## Strengths and weaknesses

### Strengths

- Supplementary documents (e.g. “EBM Fundamentals”) provide additional information and are up-to-date
- Updated daily
- Results can be filtered by “content type” (e.g. Condition, Evaluation, Procedure, etc.)
- Micromedex drug content
- Topic alerts when updates are made
- Free mobile app (free to download, but requires a subscription to use)
- Multimedia resources
- Single sign-on
- Summaries are edited by subject-matter experts
  - Editor affiliations, qualifications, and conflicts of interest are also stated

### Weaknesses

- Lacks advanced search functions (e.g. truncation, adjacency/proximity, frequency)
- High cost to access

## Currency

DynaMed was created in 1995 by Brian Alper, the former Chief Medical Knowledge Advisor at EBSCO [6]. Although DynaMed is 20 years old, the information it provides is evidence-based, updated daily, and maintained by subject matter experts [7].

## Comparison with similar products

BMJ Best Practice is a similar point-of-care tool with comparable features to DynaMed. These similarities and differences are captured in Table 1. Comparisons were made using the topic overviews for “Alcohol Use Disorder” on October 7, 2024.

**Table 1:** Comparison of DynaMed and BMJ Best Practice

	DynaMed	BMJ Best Practice
**Owner**	EBSCO Information Services	BMJ Group
**Content**	Overview and Recommendations Evaluation Management Background information Description Also Called Definitions History and Physical Clinical Presentation History Physical Diagnosis Diagnostic criteria Testing overview Management Treatments Diet Medications Consultations Complications Prognosis Prevention and Screening Guidelines and Resources Patient information References	Overview Summary Theory Epidemiology Aetiology Case history Diagnosis Approach History and exam Investigations Differentials Criteria Screening Management Approach Treatment algorithm Emerging Prevention Patient discussions Follow up Monitoring Complications Prognosis Resources Guidelines Images and videos References Patient information Calculators Evidence
**Additional Support**	DynaMed User Guide Includes video tutorials, and text and photo guides 24/7 customer support available via toll-free number Newsletters	BMJ Best Practice Website UserGuide BMJ Best Practice Podcast [8] Customer support available via phone or email
**Update Frequency**	Daily	Daily
**App Availability**	Yes	Yes
**Downloadable**	Downloadable as a PDF	Downloadable as a PDF
**Multi-Language Support**	15 languages to choose from	2 languages to choose from
**Ease of Use**	Very easy and intuitive to use	Very easy and intuitive to use
**Search Functionality**	Basic search	Basic search
**Personal Accounts**	Yes	Yes
**Individual Subscription Costs ($CAD)**	Students: $209/year [5] Clinicians: $552/year [9]	Physicians: $390/year Other health professionals: $289/year Physicians in training: $224/year Students: $145/year [10]
**Institutional Subscription**	Yes	Yes
**Free Trial Options**	Institutional free trial Must be requested Individual free trial is available	Institutional free trial only
**Mobile App Ratings**	App Store: 4.9/5 stars Based on ~3300 ratings Google Play: 4.3/5 stars Based on ~1770 ratings	App Store: 4.8/5 stars Based on 538 ratings Google Play: 4.8/5 stars Based on ~4320 ratings

Another option for users is the point-of-care tool UpToDate, which contains similar topic coverage and overviews as DynaMed and BMJ Best Practice. UpToDate is owned by Wolters Kluwer. Subscription costs vary by profession (e.g. physicians: USD $579.00/year) [11].

## Conclusion

DynaMed provides users with evidence-based information and serves as a useful tool for anyone seeking fast and reliable information and answers to clinical questions. DynaMed is easy to use and navigate, with its combination of speciality topics to choose from, and a basic search bar. The information included in DynaMed is thorough and of high quality and is maintained and updated by subject matter experts. Compared to other point-of-care tools, such as BMJ Best Practice and UpToDate, DynaMed offers similar features, including topic overviews and guidelines and is accessible online and as a mobile application–albeit, at a higher price point.
